# Recommendations for Competency in Allergy Training for Undergraduates Qualifying as Medical Practitioners: *A Position Paper of the World Allergy Organization*

**DOI:** 10.1186/1939-4551-2-8-150

**Published:** 2009-08-15

**Authors:** Paul C Potter, John O Warner, Ruby Pawankar, Michael A Kaliner, Sergio Del Giacco, Lanny Rosenwasser

**Affiliations:** 1 Allergy Diagnostic and Clinical Research Unit, University of Cape Town Lung Institute and Groote Schuur Hospital, Cape Town, South Africa; 2 Department of Paediatrics, Imperial College, Wright Fleming Institute, St. Mary's Hospital Campus, London, UK; 3 Nippon Medical School, 1-1-5 Sendagi, Bunkyo-ku, Tokyo, Japan; 4 Institute for Asthma and Allergy, Wheaton, MD; 5 Policlinico Universitario, Department of Medicine, Cagliari, Italy; 6 Pediatric Immunology Research, Children's Mercy Hospital & Clinics, Kansas City, MO; 7 Correspondence to: Karen Henley (e-mail: khenley@worldallergy.org)

**Keywords:** Undergraduate Medical Education, Training and Competencies in Allergy

## Abstract

The Council acknowledges specific comments from: The American Academy of Allergy, Asthma and Immunology (AAAAI) (Amal H Assa'ad); The American College of Allergy, Asthma and Immunology (ACAAI) (Mark Dykewicz, D. Betty Lew, Bryan L. Martin); The Argentine Association of Allergy and Immunology (Ledit RF Ardusso); The Argentine Society of Allergy and Immunopathology (Estrella Asayag); The Australasian Society of Clinical Immunology and Allergy (ASCIA) (Jill Smith); The British Society for Allergy and Clinical Immunology (Stephen Durham); The Brazilian Society of Allergy and Immunopathology (Nelson Rosario); The Bulgarian Society of Allergology (Vasil Dimitrov); The Canadian Society of Allergy and Clinical Immunology (CSACI) (Richard Warrington); The Chilean Society of Allergy and Immunology (Jessica Salinas); The Chinese Society of Allergology (Zhang Hongyu, Yin Jia); The Czech Society of Allergology and Clinical Immunology (Jiri Litzman); The Danish Society of Allergology (Lone Winther, Peter Plaschke); The Egyptian Society of Allergy and Clinical Immunology (Kamal Maurice Hanna); The Egyptian Society of Pediatric Allergy and Immunology (Yehia El-Gamal); The German Society for Allergy and Clinical Immunology (Thilo Jakob, Claus Bachert, Bernhard Przybilla); The Hungarian Society of Allergology and Clinical Immunology (Kristof Nekam); The Icelandic Society of Allergy and Clinical Immunology (Björn R. Lúðvíksson); The Italian Association of Territorial and Hospital Allergists (Riccardo Asero); The Italian Society of Allergy and Clinical Immunology (Luigi Fontana); The Japanese Society of Allergology (Sankei Nishima); The Korean Academy of Asthma Allergy and Clinical Immunology (Joon Sung Lee, Hae-Sim Park); The Latvian Association of Allergists (Ieva Cirule); The Lebanese Society of Allergy & Immunology (Fares Zaitoun); The Mongolian Society of Allergology (S. Munkhbayarlakh); The Allergy and Clinical Immunology Society (Singapore) (Chng Hiok Hee); The Allergy Society of South Africa (Sharon Kling); The Spanish Society of Allergy and Clinical Immunology (Tomás Chivato); The Swiss Society for Allergology and Immunology (SSAI-SGAI) (Beat A. Imhof, Andreas Bircher); The Allergy and Immunology Society of Thailand (Pakit Vichyanond); The Turkish National Society of Allergy and Clinical Immunology (Omer Kalayci); and The Venezuelan Society of Allergy, Asthma and Immunology (Luis F Sarmiento).

## Introduction

The global increased prevalence of allergy is such that between 20-30% of the world's population now suffers from some form of allergic disease, with considerable and continuing increases in prevalence over the last three decades [1]. Although the specialty of allergy is practiced and recognized in most developed countries, even some developed countries lack adequate resources to manage the local burden of allergic disease. In many developing countries there are few or no allergy specialists due to either the prevailing healthcare infrastructure, to socio-economic reasons, and/or to the lack of recognition of allergy as a clinical specialty. There is often minimal or no inclusion of allergy education/training in the undergraduate medical curriculum, and this shortfall must be addressed if the increasing burden of allergic diseases is to be managed.

The majority of patients with common allergic diseases around the world are treated by primary care physicians, and not by trained specialists. However, a lack of appropriate education and training in allergy at the undergraduate level leaves many medical graduates with low baseline knowledge and skills in the science and practice of allergy. In addition, because it is a relatively new discipline, education and training in allergy in medical schools has lagged behind scientific and clinical developments in this field, and there are few allergy specialists available to teach this multidisciplinary subject. This phenomenon is described by the World Health Organization as the knowledge/practice gap. Unless allergy training is included as an essential part of undergraduate medical education at the clinical level, many physicians will qualify with inadequate competency to manage the diagnosis and treatment of allergic diseases at the primary care level. Thus, a cycle of lack of basic knowledge about the most common allergic diseases, lack of recognition of allergic disease at the clinical level, and inadequate knowledge and skills in the diagnosis and treatment of allergic diseases will be perpetuated.

To help break this cycle the World Allergy Organization (WAO) presents broad guidelines for the curriculum of education and training of medical students in the immune mechanisms of allergic responses, and the commonest manifestations of clinical allergy. Inclusion of these educational guidelines into curriculum development will provide medical graduates with the basic knowledge required to recognize and treat common allergic diseases during postgraduate training or as a general practitioner (care level 1), and the knowledge of when to refer the more complex problems to appropriate organ-based or allergy specialists (care levels 2 and 3) [2]. These guidelines outline optimal curriculum content, and are offered for consideration and modification to meet local needs and healthcare provision structures. Although certain immunodeficiency states may accompany allergies or may need to be considered in the differential diagnosis of allergic diseases, this document is not intended to provide a comprehensive guideline on the teaching of immune deficiencies to medical students.

## Broad Objectives of the WAO Guidelines for Medical Student Training in Allergy

The objectives are concordant with the recommendations made by the WAO in the 2008 WAO Position Statement "Requirements for Physician training in Allergy: Key clinical competencies appropriate for the care of patients with allergic or immunologic diseases"[2] and align with competency at the first level of care. This represents only the first step underlying any training program for physicians who will be seeing patients with allergy.

## Levels of Competence

Levels of competence required for medical school graduates are divided into 2 main categories as follows:

I. Threshold Knowledge

a. An in-depth understanding

b. Familiar with principles

c. Aware of principles

II. Practical Skills

a. Competent to perform and fully understand the scientific basis and clinical value

b. Witnessed its use in practice and full understanding of scientific basis and clinical value

c. Familiar with principles and scientific basis

## Knowledge

1. Knowledge of the basic science and normal physiology of the immune response. Knowledge at this level should include major mechanisms of host defense (innate and adaptive); antigen presentation and sensitization; the nature of humoral and cellular responses; the roles of leukocytes, especially eosinophils, basophils, mast cells, dendritic cells, T cells, B cells, stromal cells, mucosal epithelial cells, cytokines, and chemokines. In particular, an understanding of Gell and Coombs I-IV hypersensitivity reactions is essential as are an understanding of the late phase allergic response [**Ia**] and some knowledge of inhalant, food, drug, insect and latex allergens [**Ib**].

2. Knowledge of the global allergy epidemic, the epidemiology of allergic diseases and the likely gene-environment interactions that affect prevalence [**Ic**].

3. Adequate clinical knowledge about the most common allergic diseases and the WAO classification of allergic disease, including allergic rhinitis, allergic conjunctivitis, rhinosinusitis, asthma, urticaria, hereditary and acquired angioedema, atopic eczema, allergic contact dermatitis, food allergy, insect venom allergy, anaphylaxis, drug allergy, occupational allergy, and eosinophilic enteropathies [**Ib**].

4. Adequate understanding about comorbidities of allergic diseases and the concept of the allergic march, including the "reverse" atopic march [**Ib**].

5. Adequate theoretical knowledge of the mechanisms underlying the interpretation of the basic diagnostic allergy tests [**IIb**], patch tests, skin prick tests [**IIb**], and serological tests [**IIb**] for total and specific IgE, and an understanding of spirometry [**IIb**] and peak flow measurement of lung function. Such training need not include competency in performing skin tests or pulmonary function tests, but in some countries training in anterior rhinoscopy or nasal endoscopy could be offered [**IIc**].

6. Knowledge of evidence based therapeutic strategies (indications, mechanism of action, efficacy, benefits, side effects, and contraindications) for treating allergic diseases, such as antihistamines, oral steroids, inhaled and intranasal steroids, and anti-IgE [**Ib**]; limitations of injected steroids; leukotriene and anti-IgE modifiers; theophylline; anticholinergics; bronchodilators; cromones; adrenaline/epinephrine [**Ia**]; allergen specific immunotherapy (sublingual and/or subcutaneous specific allergen immunotherapy) [**Ib**], and anti-IgE therapy. Knowledge of adverse medication reactions, and differentiating adverse from allergic drug reactions, is clinically critical [**Ic**].

7. Adequate training to recognize the severity and persistence of allergic diseases and the importance of referral to an allergy specialist for evaluation and initiation of treatment before the disease advances to a severe or life-threatening stage. This will also include referral for anti-IgE therapy and in some countries would apply to the initiation of specific allergen immunotherapy [**Ic**].

8. Knowledge of the concepts of primary and secondary immune responses, and response to vaccines and infection [**Ib**].

9. Knowledge of the clinical manifestations of anaphylaxis, concepts of emergency treatment, and knowledge of patient-initiated self-medication with injectable epinephrine [**Ia**].

10. Understanding of the elements of primary and secondary prevention of allergic disease [**Ic**].

## Different Formats for Teaching Allergy to Medical Students

Allergy can be taught in a number of components of the curriculum and can be included among the subjects taught in Medicine, Pediatrics, Microbiology, and Immunology, or as a component of organ-based specialty teaching, for example, dermatology, ENT, internal medicine, and ophthalmology. If allergy is taught as a component of different organ-based specialties, a cohesive program should be developed to ensure that all major allergic disorders are covered. In some countries such programs could be co-ordinated and be established by Allergy departments.

Teaching allergy as part of systematic undergraduate training and as problem solving around cases, are not mutually exclusive and can be combined. Each country can attempt to include the essential topics into existing curricula in different medical school training programs.

### A. Systematic Undergraduate Education and Training in Allergy

In this approach, students receive their teaching in two phases:

#### Part I: Basic science

• We assume that students have already undergone education in the anatomy [**Ia**], physiology and biochemistry relevant to the target organs of allergic manifestations--lungs (eg, lung volumes, control of respiration, and adrenergic and histamine receptors) [**Ia**], skin (eg, barrier function), nose, eye, gut, and elements of statistics [**Ic**].

• It is important that students are made aware of the important indigenous allergens in their country and region and in different countries around the world [**Ic**].

Two specific general lectures:

1. *General overview (mast cells, basophils, T and B cells, eosinophils, and so forth) *[**Ib**].

2. *Mechanism of organ-specific responses, for example, Early/Late phase responses in skin, nose, eyes, lung, and systemic allergic reactions *[**Ia**].

## Additional Topics for Clinical Seminars

1. Trigger factors and risk factors: A general knowledge of the common allergens [house dust mite, pollens (grass, tree, weed), cat and dog allergens, molds, insects/venoms, foods, drugs] and other triggers of symptoms such as indoor and outdoor pollutants. Knowledge about regional inhalant sensitivities. Knowledge about allergen avoidance and environmental control measures [**Ib**].

2. Pharmacology: In addition to what is taught in general pharmacology, additional information on the anti-inflammatory roles of pharmacotherapeutic anti-allergic agents, safety issues, and indications and contraindications of antihistamines, steroids (inhaled, nasal, and oral), leukotriene modifiers, bronchodilators, anticholinergics, cromones and theophyllines [**Ia**], allergen specific immunotherapy [**Ib**], and anti-IgE [**Ic**].

3. Immunology of the allergic response: Early and late phase reactions [**Ia**], basic information on cells and mediators involved in the allergic response, immunologic basis for allergy tests, mechanisms of pharmacotherapy, mechanisms of immunotherapy [**Ib**].

4. WAO classification of allergic disease [**Ib**] [3].

### Part II: Clinical allergy training (with formal lectures, clinical tutorials, ward rounds, case studies, clinic attendance, clinico-pathology conferences)

Three specific clinical allergy lectures:

1. IgE and anaphylaxis.

2. Importance of allergy in asthma, rhinitis, eczema and occupational diseases, and their allergic comorbidities.

3. Treatment strategies emphasizing allergen avoidance, patient education, and immunotherapy in addition to pharmacotherapy.

## Suggested Topics to be Covered in Clinical Lectures

1. A brief knowledge about the global allergy epidemic and epidemiology of allergic diseases.

2. Clinical presentations of asthma, rhinitis, sinusitis, allergic conjunctivitis, atopic eczema, urticaria, food allergy, eosinophilic enteropathies, drug allergy, insect/venom allergy, anaphylaxis, and occupational allergies (eg, latex), using the WAO nomenclature for allergy [**Ib**] [3].

3. How in vivo and in vitro diagnostic tests are ordered and interpreted: Skin prick tests, serum specific IgE antibodies [**Ib**], patch tests [**Ic**], peak flow measurements [**Ia**], and spirometry [**Ib**]. Instruction on the precautions, techniques, indications, and limitations of each test should be included.

4. Basic understanding of the value of skin prick tests [**Ia**], patch tests [**Ic**], serum IgE specific antibody tests [**Ib**], peak flow measurements [**Ia**] and spirometry [**Ib**], and the quality of such tests and their limitations [**Ic**]. Management plans including:

• Allergen avoidance, knowing when it is necessary

• Indications for pharmacotherapy with steroids, antihistamines, antileukotrienes, bronchodilators, anticholinergics, theophylline, adrenaline/epinephrine auto-injectors, allergen specific immunotherapy and anti-IgE [**Ib**]

• Inhaler/spacer techniques [**Ia**]

• Drug interactions and contraindications [**Ic**]

5. Resources for patients with allergies--self help groups, Medic Alert and so forth [**Ic**].

6. Knowledge of what an Allergist can offer [4] and when to refer to a Specialist [**Ic**].

### B. Problem Based Teaching/Problem Solving Around Case Studies

At least 4 cases are recommended and study of on-line cases such as those provided on the WAO Web site http://www.worldallergy.org. In this format, utilizing a case history with multiple allergic manifestations as indicated in case 1 (below), it is possible to teach the basic sciences of allergy (as itemized in section B part 1) and clinical disease presentation, and encourage correlation between the two, and also teach pharmacotherapeutics. The combination of cases should cover the common allergens, resources for patients, WAO nomenclature for allergy,[3] and epidemiology of allergic diseases.

1. A child or an adult who has demonstrated the allergic march from atopic eczema and food allergy to asthma and allergic rhinitis with a strong family history of atopy.

2. Insect venom hypersensitivity to include mechanisms, signs and symptoms, diagnosis, and action plans for managing anaphylaxis, immunotherapy (theory and evidence for efficacy and side effects), and other treatment options.

3. Latex/food allergy or pollen/oral allergy syndrome to illustrate allergenic cross reactivity, avoidance strategies, and pharmacotherapy.

4. Urticaria and angioedema; differential diagnosis, investigations, and management.

5. A case study on anaphylaxis and emergency treatment (eg, use of epinephrine).

6. A case study of acute, severe asthma with respiratory distress that focuses on the differential diagnosis of an asthma exacerbation and the interaction between infection and allergy.

## Assessment of Competence in Allergy for Medical Students

Assessment should be made by written and oral examination using either the "problem based approach" or the "systematic teaching approach." Recommended levels of competence are marked in the syllabus.

1. Assessment of Part 1. Basic sciences such as pharmacology, physiology, and immunology should be conducted by written or multiple choice examination and could be examined within the undergraduate Pathology/Pharmacology Semester examinations.

2. Assessment of Part 2. Clinical allergy competence should be conducted by written and oral examination:

a. Where a medical student is given an "allergy case" and is asked to examine, diagnose, and then make a case presentation, after which a series of clinical questions are posed around the case (extended matching and/or multiple choice).

b. Patients with allergies, for example, atopic eczema, asthma, rhinitis, sinusitis, food allergy, insect/venom allergy could also be included in the clinical cases for the in-course and final evaluations of the students for competence in Medicine and Pediatrics. Assessments could be in the form of long cases or more reproducibly by miniCex (mini-clinical evaluation exercise), OSCE stations and/or PACEs (Practical Assessment of Clinical Examination Skills).

### Resources

It is important that textbooks suitable for undergraduate training in allergy are made available to the medical students, as well as electronic learning and on-line teaching modules.

For example:

a. Roitt's Essential Immunology. IM Roitt, PJ Delves, SJ Martin, D Burton. Blackwell Publications, 2006.

b. Cellular and Molecular Immunology by Abbas and Lichtman, publisher Saunders, 6th edition 2007.

c. Allergy 3rd Edition, ST Holgate, MK Church, L Lichtenstein ISBN: 13: 978 0 323 03227 8.

d. Allergies and their Management. RS Walls. MacLennan and Petty, Sydney, Elsevier (Australia), 1997.

e. Lecture Notes in Immunology. WG Reeves, I Todd. Blackwell Scientific Publications, 3rd edition, 2000.

f. A Color Atlas of Pediatric Allergy; JO Warner and WF Jackson. Wolfe, 1994.

g. Prevention of Allergy and Allergic Asthma. World Allergy Organization Project Report and Guidelines. Eds, S.G.O. Johansson and T. Haahtela. In: Chemical Immunology and Allergy, Vol. 84, 2004. Basel, Switzerland: Karger. Summary available at http://www.worldallergy.org/professional/who_paa2003.pdf. Eds, J Ring et al.

h. WAO GLORIA Educational Modules (covering major topics in allergy, eg, Asthma, Rhinitis, Eczema, Drug Allergy, Anaphylaxis, Urticaria, Allergic Conjunctivitis, Sinusitis and Nasal Polyposis.) Available at http://www.worldallergy.org/educational_programs/gloria.

i. The Allergic Diseases Resource Centre of the WAO Web site- http://www.worldallergy.org/adrc/ includes numerous educational synopses that provide suitable reading for students.

## Appendix

List of topics which medical students should be familiar with to be competent in allergy when graduating as a medical doctor.

### 1. Anatomy [Ia]

Structure: Upper airway, lower airway, eyes, gastrointestinal tract, skin.

### 2. Physiology [Ib]

Haemodynamics, blood pressure, lung function, lung volumes, nasal physiology, gastro-intestinal function and circulation, skin physiology and barrier function.

### 3. Immunology [Ib]

Immunology of the allergic response, mast cells, basophils, eosinophils, T Cells, regulatory T cells, IgE, cytokines IL2, IL3, 4, 5, 10, TH1 + TH2 pathways, TH17 cells, dendritic cells/antigen presenting cells. B cells, plasma cells, immunoglobulin production, class switching, cellular and humoral immune responses to proteins and small chemical compounds (haptens) [Ib-Ic]. Regional common inhalant allergens [Ib], common cross reactivities (pollen/fruit etc) brief knowledge of regional aerobiology [Ic], food allergens, occupational allergens (eg latex) [Ib], mechanisms of immunotherapy. Markers of allergic inflammation (eosinophil cationic protein, eosinophils, nitric oxide) [Ic].

### 4. Diagnostics

Mechanisms and interpretation of validated allergy skin prick tests [Ia], mechanisms of specific serum IgE tests [Ib]. Cut off values, predictive values. Total IgE values. New developments in diagnostics. Interpretation of simple lung function tests (PEFR and spirometry) [Ia] and normal ranges, and rhinometry [Ic]. Allergen patch tests [Ic]. An understanding that some tests have no scientific basis or validation (eg IgG for food and other complementary approaches, Vega testing) [Ic].

### 5. Clinical Entities

Asthma [**Ia**], allergic rhinitis [**Ia**], nonallergic rhinitis [**Ib**], acute and chronic sinusitis [**Ib**], atopic eczema **[Ia]**, food allergy [**Ia**], oral allergy syndrome [**Ib**], occupational asthma, occupational allergies, urticaria and angioedema [**Ib**], insect/venom allergy [**Ib**], allergic contact dermatitis [**Ib**], drug allergies [**Ib**], co-morbid diseases of allergy (sinusitis, otitis). Differential diagnosis of allergies (eg mastocytosis, allergic alveolitis) [**Ic**]. Hereditary and acquired C1 esterase inhibitor deficiency [**Ic**]. Knowledge about availability of management guidelines, where to find them, and key points for general practitioners. Global Initiative for Asthma (GINA) [**Ic**] and Allergic Rhinitis and its Impact on Asthma (ARIA) [**Ic**] (or national) guidelines. The evidence base for non-pharmacological treatment (eg, house dust mite avoidance).

### 6. Pharmacotherapeutics

Antihistamines (first and second generation; oral and intranasal) [**Ia**]

Glucocorticosteroids (topical and oral) [**Ia**]

Short-acting and long-acting bronchodilators [**Ia**]

Anticholinergics [**Ib**]

Theophylline [**Ia**]

Leukotriene antagonists [**Ia**]

Monoclonal anti-IgE [**Ic**]

Immunotherapy: Subcutaneous and Sublingual [**Ib**]

Pharmacoeconomics [**Ic**]

Drug interactions [**Ic**]

Adverse effects of chronic medication [**Ic**]

Monitoring growth in children and adverse events in all patients on steroids [**Ia**]

The role of specialized interventions, eg anti-IgE therapy [**Ic**]

Future potential for cytokine management of allergic diseases [**Ic**]

Emerging concepts of pharmacogenetics [**Ic**]

Calcineurin inhibitors [**Ic**]

### 7. Lines of Communication

When to refer to a specialist [**Ib**]

How to use WAO and other resources in Allergy (online) [**Ic**]

Patient resources/support groups (on-line) [**Ic**]

Resources of the National Allergy Societies in the region [**Ic**]

## End Notes

With special recognition of the contribution of Karen Henley, staff liaison to WAO Specialty and Traning Council
